# A Comparative Study on Imputation Techniques: Introducing a Transformer Model for Robust and Efficient Handling of Missing EEG Amplitude Data

**DOI:** 10.3390/bioengineering11080740

**Published:** 2024-07-23

**Authors:** Murad Ali Khan

**Affiliations:** Department of Computer Engineering, Jeju National University, Jeju 63243, Jeju-do, Republic of Korea; muradali@stu.jejunu.ac.kr

**Keywords:** machine learning, deep learning, imputation, big data, clinical data, EEG, PhysioNet, CHB-MIT, transformer, TabTransformer, attention

## Abstract

In clinical datasets, missing data often occur due to various reasons including non-response, data corruption, and errors in data collection or processing. Such missing values can lead to biased statistical analyses, reduced statistical power, and potentially misleading findings, making effective imputation critical. Traditional imputation methods, such as Zero Imputation, Mean Imputation, and k-Nearest Neighbors (KNN) Imputation, attempt to address these gaps. However, these methods often fall short of accurately capturing the underlying data complexity, leading to oversimplified assumptions and errors in prediction. This study introduces a novel Imputation model employing transformer-based architectures to address these challenges. Notably, the model distinguishes between complete EEG signal amplitude data and incomplete data in two datasets: PhysioNet and CHB-MIT. By training exclusively on complete amplitude data, the TabTransformer accurately learns and predicts missing values, capturing intricate patterns and relationships inherent in EEG amplitude data. Evaluation using various error metrics and R2 score demonstrates significant enhancements over traditional methods such as Zero, Mean, and KNN imputation. The Proposed Model achieves impressive R2 scores of 0.993 for PhysioNet and 0.97 for CHB-MIT, highlighting its efficacy in handling complex clinical data patterns and improving dataset integrity. This underscores the transformative potential of transformer models in advancing the utility and reliability of clinical datasets.

## 1. Introduction

Missing values in EEG signal datasets present a significant challenge, potentially leading to biased results and reduced statistical power if not properly addressed. Traditional methods for handling missing values include deletion techniques and simple imputation methods. List-wise deletion removes records with missing values, preserving dataset integrity but often losing substantial data, especially with high missingness rates according to Ndifon et al. [1]. Pairwise deletion retains all available data but can lead to inconsistencies and biased estimates.

Simple imputation methods, presented by Rashid et al. [2], such as mean, median, or mode imputation, replace missing values with summary statistics of observed data. While easy to implement, these methods can distort data distribution and underestimate variability. More sophisticated approaches, like regression imputation and expectation–maximization (EM) algorithms, improve on this by leveraging relationships within the data to estimate missing values. Regression imputation uses available data to predict missing values based on a regression model, while the EM algorithm iteratively estimates missing data and model parameters until convergence, as suggested by Yu et al. [3].

Multiple imputation, introduced by Nijman et al. [4], further enhances imputation accuracy by creating several complete datasets with different imputed values and combining results to account for imputation uncertainty. This method is a gold standard due to its robust statistical properties. Another paper by Lin et al. [5] presents a voltage-controlled optical phantom for brain NIRS signal simulation, offering high stability and a broad dynamic range essential for NIRS device validation and BCI training, with potential applications in machine learning-enhanced signal analysis and data interpretation. Recent advancements in machine learning have introduced sophisticated imputation techniques, utilizing algorithms such as k-nearest neighbors (k-NN), random forests, and neural networks (Gond et al. [6] and Tavazzi et al. [7]). These methods capture complex data patterns, offering improved accuracy compared to traditional statistical techniques. However, they often require substantial computational resources and may struggle with high-dimensional data.

More recently, deep learning approaches have gained attention for their capability to handle complex imputation tasks. Variational auto-encoders (VAEs) and generative adversarial networks (GANs) have been used for data imputation, showing promise in capturing the underlying distribution of the data and providing robust imputations [8,9]. Liang et al. [10] explored the evolving stroke burden in China from 1990 to 2019, forecasting increased cases and deaths despite declining rates, emphasizing the role of big data analytics and machine learning for effective prevention and management. A sparse Bayesian learning approach for end-to-end EEG decoding is presented by Wang et al. in [11], outperforming deep learning methods on motor imagery and emotion recognition datasets, and advancing neuroscientific applications in brain–computer interfaces. Despite their effectiveness, these models can be computationally intensive and require significant expertise to implement.

In this paper, we propose a novel approach for imputing missing values using TabTransformer models, which have demonstrated exceptional performance in natural language processing and time series prediction tasks. Transformers, introduced by Shaw et al. [12], utilize self-attention mechanisms to capture dependencies across data points, making them particularly well-suited for imputation tasks involving complex and non-linear relationships between variables.

Our methodology involves systematically preparing the data, training the TabTransformer model on subsets of complete data, and iteratively predicting and filling in missing values. This approach leverages the TabTransformer’s ability to model intricate patterns and dependencies within the data, offering a robust solution for missing value imputation in clinical datasets. By building on the strengths of both traditional and machine learning-based imputation methods, our proposed methodology aims to provide a comprehensive and efficient solution to the challenge of missing data, ensuring the integrity and usability of clinical datasets for subsequent analysis and decision-making. The key contributions of this research are the following:

- Innovative Use of TabTransformer Models for Imputation: this research introduces TabTransformer architectures for predicting and filling missing values in EEG amplitude datasets, capturing complex data relationships more accurately than traditional methods.
- Systematic Data Preparation and Training Process: the methodology includes detailed steps for data preparation and training, ensuring the TabTransformer model is trained on the most informative EEG amplitude data for enhanced predictive performance.
- Comprehensive Evaluation of Imputation Performance: the Proposed Model’s performance is rigorously evaluated using multiple metrics, demonstrating substantial improvements over traditional imputation methods like Zero, Mean, and KNN Imputation.
- Verification through LSTM Model Analysis: the study employs an LSTM network to verify the imputed data effectiveness, showing that the proposed TabTransformer-based method maintains EEG amplitude data integrity and predictive power better than other techniques.
- Enhancing EEG amplitude data Integrity and Usability in Clinical Research: the proposed imputation method significantly improves the completeness and reliability of EEG amplitude datasets, supporting more accurate analyses and better decision-making in clinical research.

## 2. Literature Review

Handling missing data has been a longstanding challenge in data analysis, with numerous methods developed to address it. Traditional imputation techniques such as mean, median, and mode imputation, while straightforward, often fail to capture the underlying data distribution, leading to biased results and underestimated variability. More sophisticated methods, such as multiple imputation, have been shown to provide robust estimates by accounting for the uncertainty associated with missing data. Multiple imputation generates several plausible datasets and combines the results to produce more accurate and reliable statistical inferences [13,14].

In recent years, machine learning approaches have gained prominence for their ability to model complex relationships within data, thereby improving imputation accuracy. Methods like k-NN and random forests have been widely adopted. For example, MissForest, an iterative imputation method using random forests presented by Sundeep et al. [15], was demonstrated to outperform traditional techniques in handling mixed-type data. Deep learning models, particularly variational autoencoders (VAEs) and generative adversarial networks (GANs), have shown promise in imputing missing values by learning latent data representations [16,17]. Furthermore, Zhang et al. [18] highlight machine learning advancements that simplify the modeling and control of continuum robots, enhancing their anti-interference and generalization capabilities. Another research by Yan et al. [19] enhanced machine learning efficiency with FeMPIM, an FeFET-based processing-in-memory cell that integrates logic operations and content searching to address the Von Neumann bottleneck. Similarly, Abbasi et al. [20] used a deep multilayer perceptron neural network for real-time classification of neonatal sleep–wake states from multichannel EEG, achieving up to 83% accuracy. These models can capture complex dependencies and generate realistic imputations, significantly enhancing the quality of the imputed data.

Transformer models, initially developed for natural language processing, have recently been applied to missing data imputation due to their powerful self-attention mechanisms. These models excel at capturing long-range dependencies and intricate patterns within the data. For instance, Yildiz et al. [21] utilized transformers for time series data imputation, demonstrating superior performance compared to traditional methods. Similarly, Ayub et al. [22] applied transformers to multivariate data imputation, achieving significant improvements in imputation accuracy. Other studies have explored the use of transformers in various domains, such as clinical data by Liu et al. [23] and sensor data by Lotfipoor et al. [24], further validating their effectiveness in handling missing data. Another study by Xi et al. [25] investigates high-order brain network interactions in ADHD boys during facial emotion processing, revealing significant differences in key brain regions and suggesting implications for machine learning in understanding ADHD-related brain network complexities. CEFormer, proposed by Yin et al. [26], is a Convolution–Transformer hybrid for image feature extraction, integrating E-Attention and convolutional modules to enhance stability, convergence speed, and accuracy, achieving up to 85.0% on ImageNet1k and surpassing other models in the Mask R-CNN framework for mAP scores. Another study suggested by Zheng et al. [27] proposed a lightweight Transformer image feature extraction network using linear attention and token pruning, achieving up to a 70% reduction in computational cost while maintaining performance within acceptable margins. Our proposed methodology builds on these advancements by systematically preparing and iteratively imputing missing values using transformer models, aiming to leverage their strengths in capturing complex data patterns and dependencies.

Building on the success of transformer models in missing data imputation, recent work has explored the integration of attention mechanisms with other machine learning methods to further enhance imputation performance. For instance, hybrid models combining convolutional neural networks (CNNs) with transformers have shown potential in spatial data imputation, where CNNs capture local patterns and transformers address global dependencies presented by Shen et al. [28]. The research by Liu et al. [29] proposed a taxonomy and machine learning-based real-time classification of ECG acquisition artifacts, achieving a 90.89% recognition rate in offline experiments. Similarly, the paper by Qureshi et al. [30] presents a highly accurate, efficient neural network for real-time classification of upper-limb sEMG signals using Log–Mel spectrograms. Furthermore, a synergistic approach has been particularly effective in geospatial and environmental datasets, where both local terrain features and broader environmental trends play crucial roles [31]. Additionally, studies like that of Khan et al. [32] applied similar hybrid models to complex financial datasets, achieving improved imputation accuracy over standalone machine learning techniques. In another study, Siddiqa et al. [33] leveraged autoML to develop and compare 18 machine learning models for neonatal sleep–wake classification using multichannel EEG, achieving a maximum accuracy of 84.78% with a Random Forest estimator. Similarly, a Multi-Branch CNN by Siddiqa et al. [34] using single-channel EEG achieved 74.27% accuracy for neonatal sleep staging, highlighting the F3 channel’s effectiveness and potential for simplified, efficient sleep monitoring.

Another notable advancement is the use of graph neural networks (GNNs) in conjunction with transformers for imputing missing data in network-based structures. This approach leverages the relational information inherent in graphs, which is often overlooked by traditional imputation methods. Research by Kim et al. [35] introduced a model that combines graph attention networks with transformer architectures to effectively handle missing data in social network analysis. Zhang et al. [36] explored image-guided hematoma evacuation via the para-corticospinal tract approach, focusing on protecting the corticospinal tract to improve outcomes for patients with intracerebral hemorrhage, potentially enhancing procedural precision. This method not only preserves the structural integrity of the data but also captures the contextual relationships among nodes, resulting in more accurate imputations, presented by Shen et al. [37]. The effectiveness of this approach has also been demonstrated in the context of biological networks, where accurate data recovery is critical for the downstream analysis conducted by Feng et al. [38].

Further, the potential of reinforcement learning (RL) to optimize the sequence of imputation steps in an iterative process has been investigated. By modeling the imputation process as a decision-making problem, Rachmawan et al. [39] showed that RL algorithms can dynamically select the most appropriate imputation method based on the state of the dataset at each step. This adaptive strategy, also presented by Smith et al. [40], has shown promise in complex datasets with patterns that change over time, such as in dynamic economic models or health records. Empirical studies, such as Li et al. [41], have documented significant improvements in the stability and reliability of imputed datasets when compared to static imputation methods. A hybrid DCNN–SVM model, proposed by Awais et al. [42], achieved 93.8% accuracy in classifying neonatal sleep–wake states based on facial expressions in video. This evolving field highlights the importance of flexibility and adaptability in advanced imputation techniques, paving the way for more personalized and context-sensitive approaches to handling missing data.

## 3. Proposed Methodology

The proposed methodology for imputing missing values in EEG amplitude datasets leverages the capabilities of TabTransformer models to predict and fill in missing data iteratively. This approach ensures that the model effectively captures the intricate dependencies within the data through a series of systematic steps. After imputing the missing values using the proposed methodology and other imputation techniques like zero imputation, mean imputation, and KNN imputation, we obtain four completed EEG amplitude data using four imputation techniques. Then, these imputed data are given to the LSTM model to check the results of different imputed data for the verification of the Proposed Model.

### 3.1. Overview of the Framework

**Figure 1:**
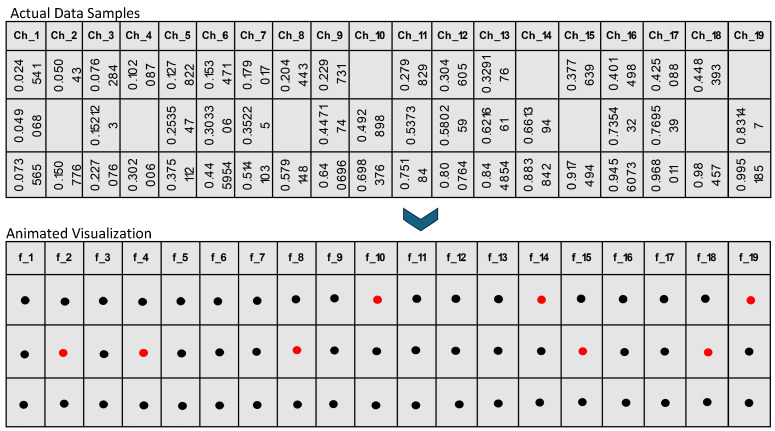
Based figure to understand the black and red dots in Figure 2’s animation procedure.

**Figure 2:**
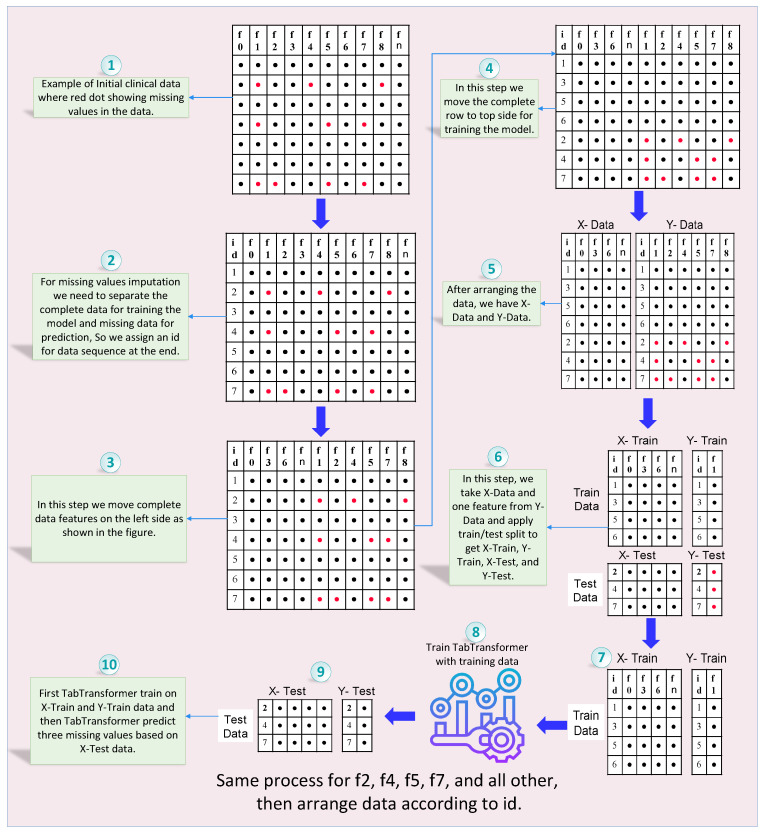
Complete process of missing values imputation using TabTransformer-based prediction mechanism.

In the proposed framework, as illustrated in Figure 1 and Figure 2, we initially prepare the dataset by identifying and marking the missing values, which are visually represented by red dots. This notation aids in clearly distinguishing between available data (black dots) and absent data points across the EEG amplitude features used in the study, as shown in Figure 1. Each row in the dataset, corresponding to a unique patient record, is subsequently assigned a unique identifier (ID). This ID is critical for maintaining the original sequence of the dataset through the various stages of the imputation process, ensuring both traceability and organizational integrity, as shown in Figure 2. This structured approach allows for precise handling and recovery of missing EEG data amplitudes, which are essential for accurate and effective analysis.

Next, we focus on identifying features with no missing values (e.g., $f_{0}$, $f_{3}$, $f_{6}$, and $f_{n}$) and repositioning these complete features to the left side of the dataset. This reorganization helps create a clear distinction between complete and incomplete data features, facilitating subsequent steps. Additionally, rows without any missing values are moved to the top of the dataset. These complete rows will serve as the primary training data for the TabTransformer model.

The EEG amplitude dataset is then divided into two parts: X-data (features) and Y-data (target with missing values). This division allows us to focus the model training on specific channel amplitude features that contain missing data. We then select a target amplitude from Y-Data that has missing values (e.g., $f_{1}$) and apply a train-test split to the X-Data and the selected Y-Data target feature. This process generates four subsets: X-Train, Y-Train, X-Test, and Y-Test, which are used for training and validating the model.

The TabTransformer model, known for its self-attention mechanisms that capture complex patterns and dependencies in the data, is trained using the X-Train and Y-Train subsets. Once trained, the model is used to predict missing values in the X-Test data. These predicted values for the target feature (e.g., $f_{1}$) are then imputed into the X-Data, effectively filling in the missing values based on the model’s predictions.

This process is iterative. The feature selection, train–test split, model training, and prediction steps are repeated for each channel amplitude feature with missing values (e.g., $f_{2}$, $f_{4}$, $f_{5}$, $f_{7}$, and $f_{8}$). In each iteration, the newly imputed values are incorporated into the updated dataset, enhancing the model’s ability to predict subsequent missing values accurately.

Finally, after all features have been imputed, the dataset is rearranged according to the initially assigned unique IDs. This ensures that the dataset’s original sequence is maintained, preserving its integrity and usability. By following this structured and detailed framework, the proposed methodology systematically addresses the challenge of missing value imputation in EEG amplitude data. It leverages the advanced capabilities of TabTransformer models to enhance data completeness and reliability, ensuring that the final imputed dataset is organized and ready for subsequent analysis and decision-making.

### 3.2. Description of EEG Amplitude Data Utilized

For the experimental analysis of imputation techniques, we utilized real EEG amplitude recordings sourced from PhysioNet and the CHB-MIT Scalp EEG Database by Shoeb et al. [43]. The columns in the dataset also shown in the table of Figure 1 are features that show the amplitude of the EEG channels. These EEG amplitudes from pediatric subjects with epilepsy were collected under controlled conditions at Boston Children’s Hospital. The dataset includes EEG signal amplitude from 23 subjects recorded using a 256 Hz sampling rate across 18 channels, following the standard 10–20 system for electrode placement. These recordings not only encompass a broad range of EEG activities but also exhibit missing values due to various reasons such as sensor disconnections, technical malfunctions, and movement artifacts. These gaps in the data are particularly prevalent and pose significant challenges, potentially leading to data loss and reducing the performance of models aimed at clinical diagnostics and analysis. The target index “Cognitive_State_Index” represents various mental states such as concentration, stress, relaxation, or engagement, providing a nuanced framework for the study. This continuous nature of the target allows for more detailed modeling, particularly in predicting and understanding the dynamics and variations in cognitive states over time.

#### Dataset Features Description

The dataset used in this study consists of EEG amplitude data collected from various channels, along with a cognitive state index. The following are more details about the features of this EEG amplitude dataset:

- Timestamp: This feature represents the time at which each EEG measurement was taken. It is a continuous variable measured in seconds, starting from zero.
- Channels (Channel_1 to Channel_19): These features represent the EEG amplitudes from 19 channels placed on the subject’s scalp. Each channel captures the electrical activity from different parts of the brain. The values are continuous and normalized, typically ranging from −1 to 1, indicating the amplitude of the EEG signal.
- Cognitive State Index: This is a continuous variable derived from EEG amplitude data to quantify the subject’s cognitive state. The index varies, indicating different cognitive states such as concentration, relaxation, or sleepiness. The exact range of this index is from approximately −2.03 to 1.78.

### 3.3. TabTransformer: Missing Values Prediction

**Figure 3:**
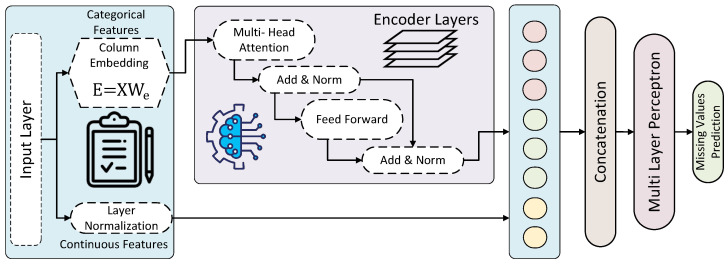
Architecture of the TabTransformer for prediction missing values.

The TabTransformer architecture shown in Figure 3, a variant of the transformer designed for tabular data, can be adapted to predict missing values in EEG amplitude data. The architecture leverages the attention mechanism to model complex dependencies within the data, leading to accurate predictions. In the coming subsection, a detailed breakdown of the TabTransformer architecture used for missing values prediction, along with its mathematical formulation, is given.

#### 3.3.1. Input Embedding and Positional Encoding

The process begins with transforming each feature vector in the tabular data into a dense representation. For numerical features, a linear layer followed by layer normalization is used, while categorical features are transformed using embedding layers. These embeddings map the features into a higher-dimensional space, facilitating the capture of underlying patterns.

Mathematically, let $X\in R^{n\times d}$ represent the input feature matrix, where *n* is the number of features and *d* is the embedding dimension. The input embedding *E* is given as

(1) $$E=XW_{e}$$

where $W_{e}$ is the embedding matrix.

Since transformers require positional information to process sequences, positional encoding is added to the embedding. Positional encoding $P\in R^{n\times d}$ is calculated using sine and cosine functions of different frequencies, as shown in Equations (2) and (3):

(2) $$P\left(\mathrm{pos},2i\right)=\sin\left(\frac{\mathrm{pos}}{10,000^{2i/d}}\right)$$

(3) $$P\left(\mathrm{pos},2i+1\right)=\cos\left(\frac{\mathrm{pos}}{10,000^{2i/d}}\right)$$

where pos is the position and *i* is the dimension index. The final input to the transformer encoder is shown in Equation (4):

(4) Z=E+P

#### 3.3.2. Encoder Layers

The transformer encoder consists of multiple layers, each comprising two main components: the multi-head self-attention mechanism and a feed-forward neural network.

- Multi-Head Self-Attention: This mechanism enables the model to focus on different parts of the input sequence by computing attention scores. The input embeddings are linearly transformed into queries *Q*, keys *K*, and values *V*, as shown in Equation (5):
  (5) $$Q=ZW_{Q},\;K=ZW_{K},\;V=ZW_{V}$$
  where $W_{Q}$, $W_{K}$, and $W_{V}$ are learned weight matrices. The attention scores are computed as in Equation (6):
  (6) $$\mathrm{Attention}\left(Q,K,V\right)=\mathrm{SoftMax}\left(\frac{QK^{T}}{\sqrt{d_{k}}}\right)V$$
  where $d_{k}$ is the dimension of the key vectors. Multi-head attention involves multiple such attention operations (heads), allowing the model to capture various aspects of the relationships between features as in Equation (7):
  (7) $$\mathrm{MultiHead}\left(Q,K,V\right)=\mathrm{Concat}\left({\mathrm{head}}_{1},\ldots ,{\mathrm{head}}_{h}\right)W_{O}$$
  where ${\mathrm{head}}_{i}=\mathrm{Attention}\left(Q_{i},K_{i},V_{i}\right)$ and *h* is the number of heads.
- Feed-Forward Neural Network: Following the self-attention mechanism, a feed-forward neural network is applied to each position independently, as shown in Equation (8):
  (8) $$\mathrm{FFN}\left(x\right)=\max\left(0,xW_{1}+b_{1}\right)W_{2}+b_{2}$$
  where $W_{1}$ and $W_{2}$ are weight matrices, and $b_{1}$ and $b_{2}$ are biases.
- Layer Normalization and Residual Connections: Each sub-layer in the transformer encoder is followed by layer normalization and residual connections, as shown in Equations (9) and (10):
  (9) $$Z^{\prime }=\mathrm{LayerNorm}\left(Z+\mathrm{MultiHead}\left(Q,K,V\right)\right)$$
  (10) $$O=\mathrm{LayerNorm}(Z^{\prime }+\mathrm{FFN}\left(Z^{\prime }\right))$$

#### 3.3.3. Pooling and Output Layers

After passing through multiple transformer encoder layers, the outputs are pooled to generate a fixed-size representation of the input sequence. Common pooling techniques include mean pooling, max pooling, and attention pooling. The pooled representation is then fed into a fully connected layer to reduce the dimensionality to a single output, corresponding to the predicted value for the missing feature.

#### 3.3.4. Model Training

The training process involves using the complete rows of data (with no missing values) to train the model. The dataset is split into training and validation sets to monitor performance and prevent over-fitting. The loss function used is MSE, measuring the difference between predicted and actual values, as shown in Equation (11):

(11) $$\mathrm{MSE}=\frac{1}{N}\sum_{i=1}^{N}{\left({\hat{y}}_{i}-y_{i}\right)}^{2}$$

where ${\hat{y}}_{i}$ is the predicted value and $y_{i}$ is the true value. An optimizer such as Adam adjusts the model parameters to minimize this loss. Once trained, the model can predict missing values in incomplete rows by inputting the available features and generating the missing values as output.

### 3.4. Verification Process of Imputed EEG Amplitude Datasets

**Figure 4:**
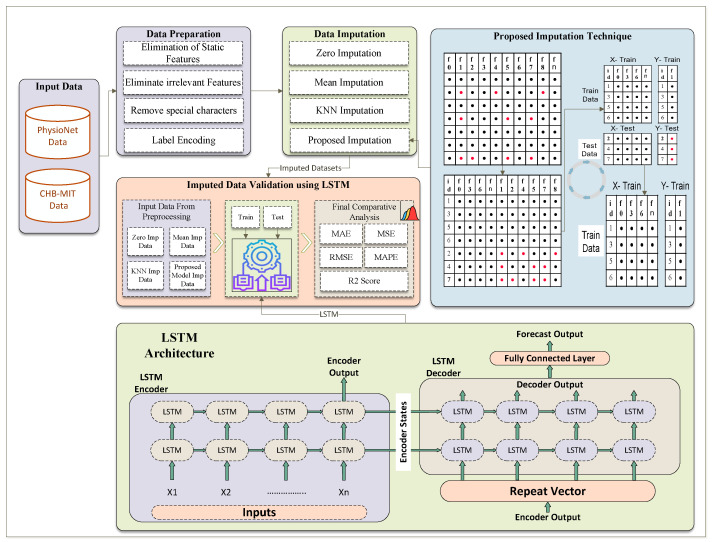
Complete process of missing values imputation using TabTransformer-based prediction mechanism.

The verification process for our imputed data involves using a Long Short-Term Memory (LSTM) network to evaluate the performance of different imputation methods. As illustrated in Figure 4, we first prepare the EEG amplitude data by eliminating static and irrelevant features and removing special characters. Following this, the data undergo imputation using four different techniques: Zero Imputation, Mean Imputation, KNN Imputation, and our Proposed Imputation method. These imputed datasets are then fed into the LSTM model to assess their effectiveness. The validation using LSTM involves the following steps:

First, we take the PhysioNet dataset, which has missing values. The PhysioNet data with missing values pass through various imputation methods.

- Zero Imputation: this method returns the zero-imputed PhysioNet data.
- Mean Imputation: this method returns the mean-imputed PhysioNet data.
- KNN Imputation: this method returns the KNN-imputed PhysioNet data.
- Proposed Imputation Method: this method returns the dataset imputed using the proposed method.

We obtained four imputed PhysioNet datasets and used them to predict the continuous target variable “Cognitive_state_Index” through an LSTM model. For training the LSTM model, we utilized the original complete rows (those without any missing values) of the EEG amplitude data. The rows with imputed values were reserved for testing the LSTM model. This approach allows us to evaluate the performance of different imputation methods by assessing how well the LSTM model, trained on complete data, predicts the target using imputed data.

We repeated this procedure for validating the CHB-MIT dataset. By comparing the LSTM results across the four imputed datasets, we can determine which imputation method most accurately reflects the original data. Superior performance of the LSTM model on a particular imputed dataset suggests that the corresponding imputation method better aligns the missing values with the actual data.

Once the imputed datasets are input into the LSTM model, we conduct a comparative analysis to determine the imputation method that yields the best results. The performance of each imputed dataset is evaluated based on five key metrics: Mean Absolute Error (MAE), Mean Squared Error (MSE), Root Mean Squared Error (RMSE), Mean Absolute Percentage Error (MAPE), and the R2 score. These metrics provide a comprehensive view of the accuracy and reliability of the imputed values. By analyzing these results, we can identify which imputation method best maintains the integrity and predictive power of the original EEG amplitude dataset. Our proposed imputation method outperforms traditional techniques, demonstrating a superior ability to preserve the underlying patterns and relationships of the data.

The LSTM network consists of an encoder–decoder architecture designed for sequence forecasting. The LSTM encoder processes an input sequence $\left(X_{1},X_{2},\ldots ,X_{n}\right)$ through multiple layers of LSTM units, capturing temporal dependencies and encoding the sequence into fixed-length context vectors (hidden states and cell states). This context vector $\left(h_{n},c_{n}\right)$ is passed to the LSTM decoder via a repeat vector layer, initializing the decoder’s LSTM units. The decoder then generates an output sequence by processing the repeated context vectors through its LSTM layers. Finally, a fully connected layer maps the decoder’s outputs to the forecasted values. The overall process ensures effective learning and prediction of time-dependent patterns in the data.

The encoder’s LSTM units update their states as Equation (12):

(12) $$h_{t},c_{t}=\mathrm{LSTM}\left(X_{t},h_{t-1},c_{t-1}\right)$$

where $h_{t}$ and $c_{t}$ represent the hidden and cell states at time step *t*. The decoder then uses the encoded context vectors to produce the output sequence Equation (13):

(13) $$Y_{t}=\mathrm{LSTM}\left(h_{t-1},c_{t-1}\right)\to \mathrm{Fully}\;\mathrm{Connected}\;\mathrm{Layer}$$

## 4. Experiment Setup

In this section, we discuss our experimental environment and the verification measures of the proposed strategy.

### 4.1. Experiment Environment

The experimental environment for this study as shown in Table 1, was meticulously configured to ensure optimal performance and reliability. The system operated on Windows 10 OS, supported by a substantial 64 GB of RAM to handle intensive computational tasks. At the core of the system was the 12th Generation Intel^®^ Core™ i9-12900K processor, running at 3.20 GHz, providing robust processing power for complex calculations and machine learning algorithms. The programming environment utilized Python 3, leveraging its versatility and extensive library support. Development was conducted using PyCharm Expert Edition, an advanced Integrated Development Environment (IDE) known for its efficiency and powerful debugging capabilities. Data management and storage were facilitated through MS Excel, ensuring organized and accessible datasets. Core libraries integral to the experiments included Keras and TensorFlow for deep learning models, NumPy for numerical computations, Requests for HTTP requests, and Seaborn and Matplotlib for data visualization. This comprehensive setup ensured a highly efficient and effective environment for conducting the study’s computational experiments.

### 4.2. Algorithm for the Proposed Model

Algorithm 1 delineates a comprehensive method for imputing missing values in EEG amplitude datasets using a TabTransformer-based model. This procedure is meticulously designed to address the complexities inherent in EEG amplitude data, ensuring that each step contributes effectively to the restoration of missing entries with high accuracy and minimal data distortion. The algorithm is structured into several key phases: data preparation, model training and prediction, data reorganization, and a final validation and comparison stage using an LSTM model. Each phase is crafted to leverage advanced machine-learning techniques that enhance the algorithm’s ability to process and improve data quality systematically.

- Data Organization and Preprocessing: Initially, the dataset *D* undergoes a preliminary process where each element $d_{ij}$ is examined for missing values. A mask $m_{ij}$ is created, where $m_{ij}=1$ indicates a missing value. This facilitates targeted operations on missing data in subsequent steps. Each row is assigned a unique identifier ID[i], preserving the original data order throughout the process. The dataset is then split into $X_{\mathrm{Data}}$ and $Y_{\mathrm{Data}}$, separating features from targets containing missing values.
- Model Training and Prediction: The algorithm iterates over each feature with missing values in $Y_{\mathrm{Data}}$. For each feature, the data are split into training and testing sets. A TabTransformer model $T_{f}$ is trained on the training set and used to predict the missing values in the testing set. These predictions, $Y_{\mathrm{Pred}}$, are then used to replace the missing values in the dataset $D^{\prime }$, effectively imputing them.
- Reorganization: Post-imputation, $D^{\prime }$ is reorganized based on the unique identifiers ID, ensuring the dataset returns to its original sequence. This step is crucial for maintaining the integrity of the dataset’s original ordering, which might be significant for subsequent analyses.
- Validation and Comparison: The final step involves validating and comparing the imputed EEG amplitude data. An LSTM model L is utilized to assess the quality of the imputed dataset $D^{\prime }$ against other datasets imputed by different methods. This comparison is conducted by evaluating metrics such as MAE, MSE, RMSE, MAPE, and the R2 score. Each dataset *r* from a collection *R* is processed through the LSTM to highlight the effectiveness of the TabTransformer-based imputation method.

**Algorithm 1:** TabTransformer-Based Missing Value Imputation for EEG Amplitude Data.    **Input:** Dataset *D* with elements $d_{ij}$, where $d_{ij}=\mathrm{null}$ indicates a missing value
    **Output:** Imputed Dataset $D^{\prime }$
    **Step 1:** Data Organization and Preprocessing
        **Initialize:** $D^{\prime }\leftarrow D$
        Mark missing values: $\forall i,j\;\mathrm{where}\;d_{ij}=\mathrm{null},\;\mathrm{set}\;m_{ij}=1$        Assign unique IDs: ID[i]←i for each row *i* in *D*        Separate features: $X_{\mathrm{Data}},Y_{\mathrm{Data}}\leftarrow \mathrm{split}\left(D,\mathrm{features}\right)$
    **Step 2:** Model Training and Prediction

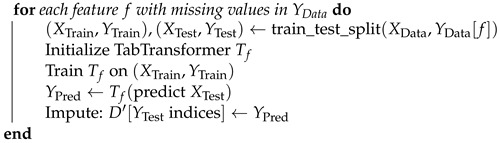

    **Step 3:** Reorganize
        $D^{\prime }\leftarrow \mathrm{sort}\left(D^{\prime },ID\right)$
    **Step 4:** Validation and Comparison
      Initialize LSTM model L
      R←listofotherimputationresults
      $R.\mathrm{append}(D^{\prime })$

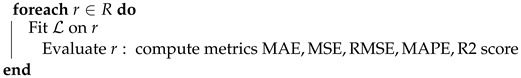



### 4.3. Verification Measures

In machine learning, regression analysis is employed to identify the relationships between dependent and independent variables. Simply put, regression is a technique used to predict continuous values such as prices, consumption levels, ratings, and more.

In clinical practice, data analysts use various performance metrics to evaluate the reliability and accuracy of predictive models. Key metrics such as MAE, MSE, RMSE, MAPE, and the R2 score provide essential insights into a model’s predictive performance. MAE measures the average magnitude of errors in a set of predictions, without considering their direction. MSE quantifies the average squared difference between the predicted and actual values, giving more weight to larger errors. RMSE adjusts MSE to the units of the output variable by taking the square root, making it more interpretable. MAPE provides a normalized error percentage, making it easier to compare performance across different datasets or models. The R2 score, or the coefficient of determination, indicates the proportion of the variance in the dependent variable that is predictable from the independent variables. By analyzing these metrics, data analysts can evaluate the effectiveness of predictive models and their potential application in clinical decision support systems and patient care management. The formulas for these performance measures are as follows:

- MAE: The Mean Absolute Error represents the average magnitude of the errors in a set of predictions without considering their direction. It is calculated in Equation (14):
  (14) $$\mathrm{MAE}=\frac{1}{n}\sum_{i=1}^{n}\left|y_{i}-{\hat{y}}_{i}\right|$$
  where $y_{i}$ are the actual values, ${\hat{y}}_{i}$ are the predicted values, and *n* is the number of observations.
- MSE: The Mean Squared Error represents the average of the squares of the errors—that is, the average squared difference between the estimated values and the actual value. It is defined as Equation (15):
  (15) $$\mathrm{MSE}=\frac{1}{n}\sum_{i=1}^{n}{\left(y_{i}-{\hat{y}}_{i}\right)}^{2}$$
- RMSE: The Root Mean Squared Error is the square root of the mean of the squared errors, providing a measure of the magnitude of the error in the same units as the response variable. It is given by Equation (16):
  (16) $$\mathrm{RMSE}=\sqrt{\frac{1}{n}\sum_{i=1}^{n}{\left(y_{i}-{\hat{y}}_{i}\right)}^{2}}$$
- MAPE: The Mean Absolute Percentage Error measures the size of the error in percentage terms. It is calculated as Equation (17):
  (17) $$\mathrm{MAPE}=\frac{100\%}{n}\sum_{i=1}^{n}\left|\frac{y_{i}-{\hat{y}}_{i}}{y_{i}}\right|$$
- R2 Score: The R2 score, or Coefficient of Determination, provides an indication of goodness of fit and, therefore, a measure of how well unseen samples are likely to be predicted by the model. The formula for the R2 score is in Equation (18):
  (18) $$R2\;\mathrm{score}=1-\frac{\sum_{i=1}^{n}{\left(y_{i}-{\hat{y}}_{i}\right)}^{2}}{\sum_{i=1}^{n}{\left(y_{i}-\bar{y}\right)}^{2}}$$
  where $\bar{y}$ is the mean of the actual values $y_{i}$.

These metrics collectively offer comprehensive insights into the accuracy and reliability of the predictive models used in the analysis.

## 5. Comparative Analysis

In this section, a critical comparative analysis of the results is conducted for both EEG amplitude datasets using different visualizations of results and different performance measures.

### 5.1. Performance Analysis for Data PhysioNet

**Figure 5:**
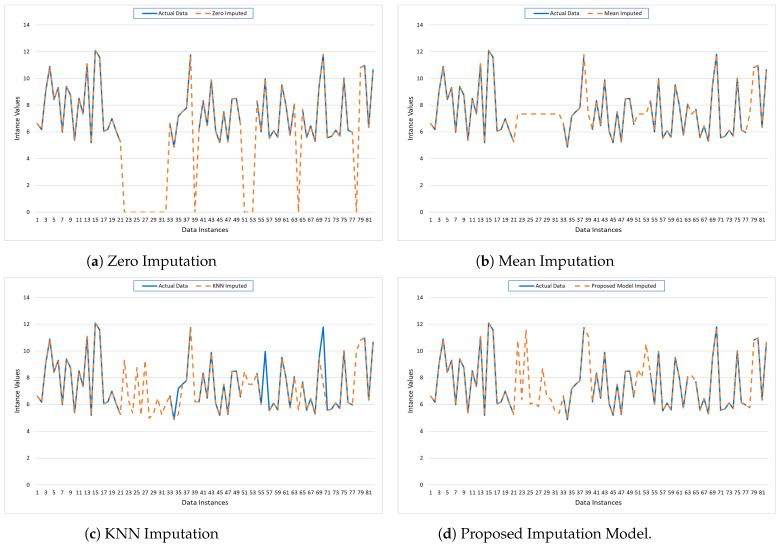
Visualization of the imputed data for PhysioNet using traditional mechanisms and the Proposed Model.

The graphs in Figure 5 illustrate the performance of four imputation models: Zero Imputation, Mean Imputation, KNN Imputation, and the Proposed Imputation model, compared to the actual data pattern of PhysioNet. Each subplot depicts the actual data (solid blue line) against the imputed data (dashed orange line) across various data instances.

- The Zero Imputation model shows significant deviations from the actual data, particularly where zeros are imputed, indicating poor performance, as shown in Figure 5a.
- The Mean Imputation model performs slightly better but still exhibits noticeable discrepancies, especially in regions where the actual data have higher variability, as shown in Figure 5b.
- In contrast, the KNN Imputation model shown in Figure 5c aligns more closely with the actual data, demonstrating improved accuracy and reduced error.
- However, the Proposed Imputation model shown in Figure 5d exhibits the best alignment with the actual data, minimizing MAE, MSE, and other errors, and achieving the highest R2 score. This close alignment underscores the model’s superior ability to accurately predict missing values, as evidenced by its minimal deviations from the actual data line.

These visual insights corroborate the quantitative metrics, confirming the Proposed Imputation model’s dominance in terms of predictive performance and accuracy.

In evaluating the performance of different imputation models, it is evident that the Proposed Imputation model significantly outperforms the others, as shown in Table 2. The Proposed Imputation model achieves the lowest MAE of 0.07 and MSE of 0.08, coupled with the lowest RMSE of 0.28 and the highest R2 score of 0.993. Additionally, it has the lowest MAPE of 0.75, indicating minimal deviation from the actual values in percentage terms. This indicates that the Proposed Imputation model has the best predictive accuracy and minimal deviation from the actual values.

In comparison, the KNN Imputation model, while performing well with an MAE of 0.08, an MSE of 0.09, an RMSE of 0.30, and an R2 score of 0.982, still falls short of the Proposed Imputation model’s superior metrics. Its MAPE of 0.82, although lower than those of Mean Imputation and Zero Imputation, is higher than that of the Proposed Imputation model. This demonstrates the efficacy of the new approach in accurately imputing missing data with higher precision.

The Mean Imputation and Zero Imputation models, with respective MAEs of 0.09 and 0.11, MSEs of 0.12 and 0.13, RMSEs of 0.35 and 0.36, and MAPEs of 0.98 and 1.05, show a marked decline in performance compared to the Proposed Imputation and KNN Imputation models. Their R2 scores, 0.965 and 0.952, respectively, also highlight their relative inadequacy in capturing the variability explained by the models. The performance gap underscores the advantages of advanced imputation techniques like the Proposed Imputation model, which leverages more sophisticated algorithms to achieve higher precision and reliability in data imputation.

Thus, the comparative analysis demonstrates that the Proposed Imputation model is the most effective among the evaluated methods, providing the best balance between low error rates, low percentage deviations, and high explanatory power.

### 5.2. Performance Analysis for Data CHB-MIT

The provided graphs illustrate the performance of various imputation techniques applied to missing values in the CHB-MIT dataset. The graphs show 81 data instances plotted on the x-axis, with their respective imputed values plotted on the y-axis. The graphs compare the actual data (solid blue line) against the imputed data (dashed orange line) for four different imputation methods: Zero Imputation, Mean Imputation, KNN Imputation, and the Proposed Model Imputation.

**Figure 6:**
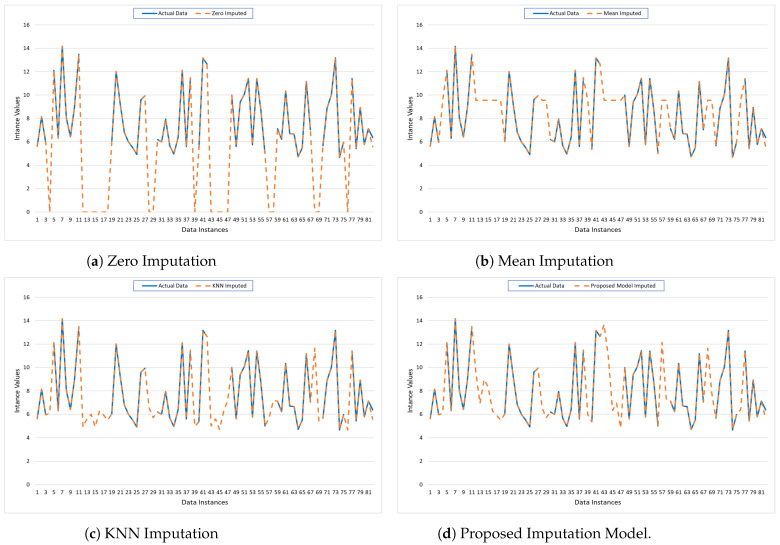
Visualization of the imputed data for CHB-MIT using traditional mechanisms and the Proposed Model.

- Zero Imputation (Figure 6a): The Zero Imputation method replaces missing values with zeros. The graph shows significant deviations between the actual and imputed values, especially in regions where the actual data have higher values. This method tends to underestimate the missing values, leading to a substantial discrepancy that is reflected in the large gaps between the two lines. Such a simplistic approach can distort the dataset’s overall pattern and may not be suitable for datasets where the values are substantially different from zero.
- Mean Imputation (Figure 6b): The Mean Imputation method replaces missing values with the mean value of the observed data. The graph indicates a better fit compared to Zero Imputation, as the imputed values hover around the average of the actual data. However, this method still fails to capture the variability and the fluctuations present in the original dataset, as evidenced by the smoother dashed line that does not align well with the peaks and troughs of the actual data.
- KNN Imputation (Figure 6c): The KNN Imputation method utilizes the nearest neighbors’ values to estimate the missing data points. This method shows a closer alignment with the actual data, capturing more of the variability and trends compared to the previous methods. The dashed line follows the actual data more closely, indicating that this technique can better preserve the underlying structure and relationships within the data, leading to more accurate imputation.
- Proposed Model Imputation (Figure 6d): The Proposed Model Imputation method, likely based on an advanced algorithm or machine learning model, demonstrates the closest alignment with the actual data. The dashed line almost overlaps with the solid line, capturing the peaks, troughs, and overall pattern of the actual data with high precision. This indicates that the Proposed Model can effectively handle the complexity and variability of the data, providing the most accurate and reliable imputation among the methods compared.

Overall, Figure 6 highlights the strengths and weaknesses of each imputation method. Zero and Mean Imputation, while simple to implement, fall short in accuracy and fail to capture data variability. KNN Imputation performs better by leveraging the structure within the data. The Proposed Model Imputation, however, shows superior performance, indicating its potential as a robust solution for imputing missing data in complex datasets. This analysis underscores the importance of selecting an appropriate imputation method to maintain data integrity and enhance the reliability of subsequent data analysis.

Furthermore, the provided Table 3 offers a comparative analysis of different imputation models for the dataset PhysioNet, showcasing their performance through various error metrics: MAE, MSE, RMSE, MAPE, and R2 score. The models assessed include Zero Imputation, Mean Imputation, KNN Imputation, and a Proposed Imputation Model.

Zero imputation performs the worst among the evaluated methods, with an MAE of 0.11, MSE of 0.13, RMSE of 0.36, and MAPE of 1.05, alongside the lowest R2 score of 0.952. This method replaces missing values with zeros, leading to significant inaccuracies and a considerable discrepancy between the imputed and actual data values. The high error metrics indicate that Zero Imputation fails to approximate the true data values accurately, underscoring its limitations in maintaining the dataset’s integrity. In contrast, mean imputation shows a slight improvement over Zero Imputation, with an MAE of 0.09, MSE of 0.12, RMSE of 0.35, and MAPE of 0.98. Its R2 score of 0.965 is higher, suggesting better alignment with the actual data. This method replaces missing values with the mean of observed data points, reducing bias but still not adequately capturing data variability. Consequently, while it is a more reliable method than Zero Imputation, it still falls short in terms of accuracy and error minimization.

Furthermore, KNN imputation significantly enhances performance, achieving an MAE of 0.08, MSE of 0.09, RMSE of 0.30, and MAPE of 0.82, with an impressive R2 score of 0.982. By utilizing the values of the nearest neighbors to estimate missing data, KNN Imputation effectively preserves the underlying structure and relationships within the data. This results in lower errors and higher explanatory power, indicating a substantial improvement over simpler imputation techniques. Moreover, the proposed imputation model delivers the best results, with the lowest error metrics: MAE of 0.07, MSE of 0.08, RMSE of 0.28, and MAPE of 0.75. The R2 score of 0.993 is the highest among the compared models, demonstrating its superior capability to explain the variance in the data. This model likely employs advanced algorithms or machine learning techniques to handle the complexity and variability of the dataset more effectively. Its outstanding performance across all metrics highlights its robustness and precision in imputing missing values, making it the most reliable method for this dataset.

In summary, while Zero and Mean Imputation methods offer basic solutions with moderate accuracy, KNN Imputation and the Proposed Model provide significantly better performance. The Proposed Model, in particular, excels in minimizing errors and maximizing explanatory power, underscoring the importance of using advanced imputation techniques to enhance data quality and integrity for subsequent analyses.

## 6. Discussion

In the evaluation of imputation techniques for clinical datasets, the proposed imputation model demonstrated superior performance across several critical metrics, including MAE, MSE, RMSE, MAPE, and R2 score. It outshines traditional imputation methods like Zero, Mean, and KNN Imputation by consistently achieving lower error rates and higher reliability in predictions.

**Figure 7:**
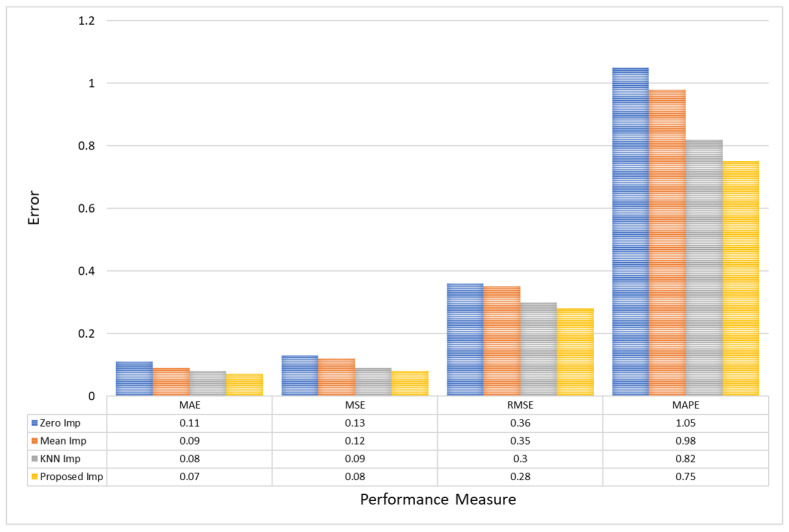
A comparative analysis of model error for PhysioNet EEG amplitude data.

Notably, for the PhysioNet dataset, the Proposed Model exhibits a 0.04 reduction in MAE compared to Zero Imputation, and a 0.3% reduction in MAPE, indicating more accurate and proportionally correct imputation. Furthermore, the RMSE of the Proposed model (0.28) versus that of Zero Imputation (0.36) suggests fewer variations and outliers in the data predictions, as shown in Figure 7.

**Figure 8:**
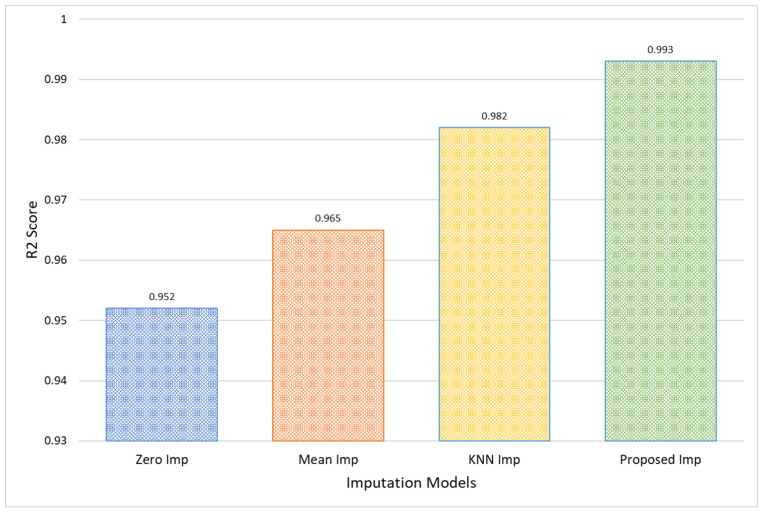
Comparative analysis in terms of R2 score for PhysioNet EEG amplitude data.

The effectiveness of the Proposed model is further validated by an R2 Score of 0.993, signifying that the model explains 99.3% of the variance within the dataset PhysioNet, a substantial improvement of 4.1% over the 95.2% accounted for by Zero Imputation, as shown in Figure 8. This high R2 value implies not only improved prediction accuracy but also enhanced capability to capture and reflect the underlying data patterns, making the Proposed Imputation model particularly valuable in clinical settings where accurate data representation is critical for decision-making and patient care.

**Figure 9:**
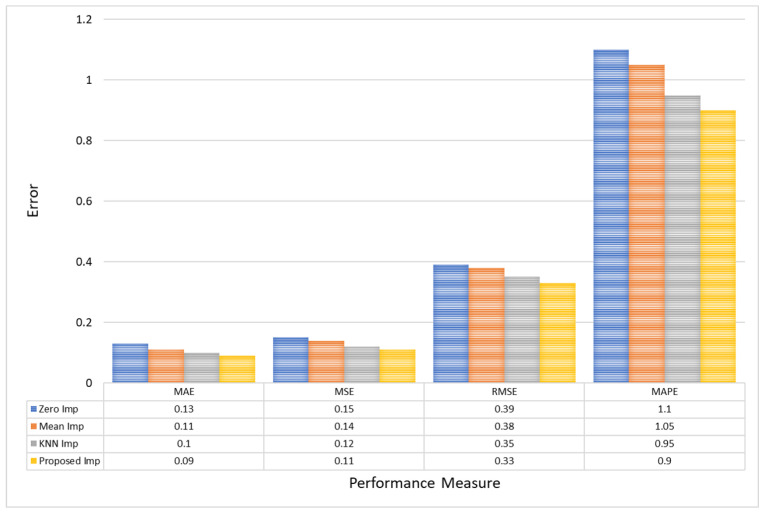
A comparative analysis of model error for CHB-MIT EEG amplitude data.

Furthermore, for CHB-MIT EEG amplitude data, the Proposed model reduces the MAE to 0.09, underscoring a decrease of approximately 0.04 from Zero Imputation’s 0.13. Similarly, the MSE sees a decrement from 0.15 in Zero Imputation to 0.11 with the Proposed model, reinforcing the precision of this method. Furthermore, the RMSE and MAPE improvements are substantial, with the Proposed model achieving the lowest values of 0.33 and 0.9, respectively, compared to Zero Imputation’s 0.39 and 1.1. This highlights the model’s ability to minimize large errors more effectively, which is crucial for maintaining reliability in clinical data interpretations, as shown in Figure 9.

**Figure 10:**
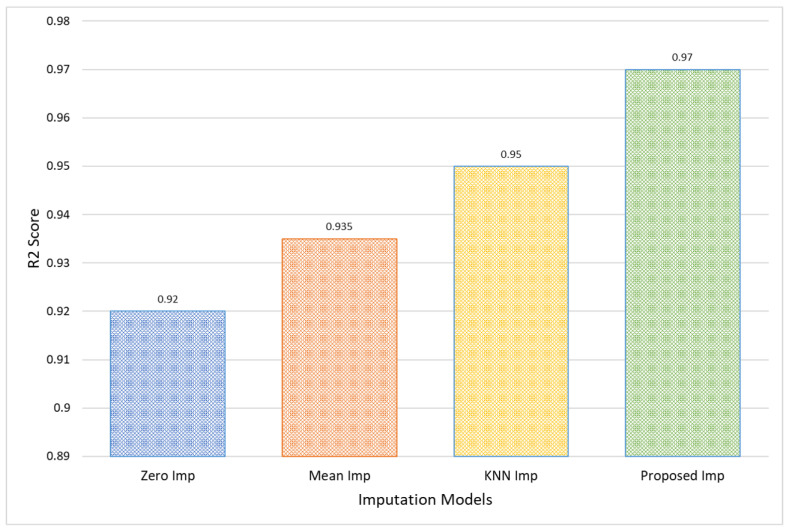
Comparative analysis in terms of R2 score for CHB-MIT EEG amplitude data.

Moreover, the Proposed Model’s R2 score of 0.97 for the CHB-MIT dataset indicates a nearly complete variance explanation by the model, which is significantly higher than that of Zero Imputation at 0.92, as shown in Figure 10. This excellent R2 score represents a near-perfect prediction capability, which not only confirms the model’s accuracy but also its consistency in different sets of data. Such a high R2 value is indicative of the model’s robustness, making it an optimal choice for clinical settings where predictive accuracy is crucial for effective decision-making.

These consistent improvements across various performance metrics validate the Proposed Model’s advanced methodology in handling the intricacies of clinical data, potentially leading to more accurate diagnoses and treatment plans.

## 7. Conclusions

Addressing missing data in clinical datasets is critical for maintaining the integrity and reliability of statistical analyses and clinical decision-making. Traditional imputation methods like Zero Imputation, Mean Imputation, and KNN Imputation have their limitations, such as bias introduction, underestimation of variability, and computational inefficiency. In response, our Proposed Imputation model leverages advanced machine learning techniques to accurately predict and impute missing values. Through rigorous evaluation using MAE, MSE, RMSE, MAPE, and R2 score metrics, we have demonstrated significant improvements over traditional methods. For instance, on the PhysioNet dataset, our model reduced MAE by 0.04 and improved MSE by 0.05 compared to Zero Imputation. The RMSE and MAPE were notably lower, indicating better maintenance of data integrity and variability. Moreover, achieving R2 scores of 0.993 for PhysioNet and 0.97 for CHB-MIT showcases the model’s ability to explain variance effectively. These advancements underscore the Proposed Imputation model’s capability to handle complex clinical data patterns, enhancing data accuracy and reliability for improved clinical research and decision-making.

## 8. Future Research Suggestions

Moving forward, it would be beneficial to expand the validation of the Proposed Imputation model across more diverse clinical datasets, including those with varying types, sizes, and complexities of missing data. Exploring its applicability in real-world clinical settings and integrating it with electronic health record systems could further validate its effectiveness and utility. Additionally, incorporating recent advancements in artificial intelligence, such as deep learning techniques, could enhance the model’s predictive accuracy and efficiency. Investigating the ethical implications of automated data imputation, particularly in sensitive healthcare contexts, is also crucial to ensure that the use of such technology adheres to the highest standards of patient confidentiality and data integrity. These steps will not only refine the model but also broaden its impact, making it a more versatile tool in the healthcare data management arsenal.

## Figures and Tables

**Table 1:** System configuration and description.

Component of the System	Details
Operating System	Windows 10 OS
Main Memory	64 GB RAM
CPU	12th Gen Intel(R) Core(TM) i9-12900K 3.20 GHz
Programming Language	Python 3.7
Development IDE	PyCharm Expert Edition 2022.1.1
Database	MS Excel Professional Plus 2019
Core Libraries	Pandas=2.2.2, Scikit-Learn=0.20.0, Keras-2.9.0, TensorFlow=2.9.1, Seaborn=0.12.2, Matplotlib=3.5.3, etc.

**Table 2:** Comparison of different imputation models for PhysioNet EEG amplitude data.

Models	MAE	MSE	RMSE	MAPE	R2 Score
Zero Imp	0.11	0.13	0.36	1.05	0.952
Mean Imp	0.09	0.12	0.35	0.98	0.965
KNN Imp	0.08	0.09	0.30	0.82	0.982
Proposed Imp	0.07	0.08	0.28	0.75	0.993

**Table 3:** Comparison of different imputation models for CHB-MIT EEG amplitude data.

Models	MAE	MSE	RMSE	MAPE	R2 Score
Zero Imp	0.13	0.15	0.39	1.10	0.920
Mean Imp	0.11	0.14	0.38	1.05	0.935
KNN Imp	0.10	0.12	0.35	0.95	0.950
Proposed Imp	0.09	0.11	0.33	0.90	0.970

## Data Availability

The data supporting this study’s findings are available from the corresponding author upon request.
